# Profil épidémiologique des pathologies respiratoires chez l’enfant à l’Hôpital d’Enfants de Rabat, Maroc

**DOI:** 10.11604/pamj.2017.28.288.13405

**Published:** 2017-12-04

**Authors:** Ilham Benchekroun, Mohamed El Mahdi Boubkraoui, Nour Mekaoui, Lamia Karboubi, Chafiq Mahraoui, Badr Sououd Benjelloun Dakhama

**Affiliations:** 1 Service des Urgences Médicales Pédiatriques, Hôpital d’Enfants de Rabat, Maroc; 2 Faculté de Médecine et de Pharmacie de Rabat, Université Mohammed V de Rabat, Maroc; 3 Service de Pneumoallergologie et Infectiologie Pédiatriques, Hôpital d’Enfants de Rabat, Maroc

**Keywords:** Pathologies respiratoires, enfant, épidémiologie, Maroc, Respiratory diseases, child, epidemiology, Morocco

## Abstract

**Introduction:**

Les pathologies respiratoires représentent un motif fréquent d'hospitalisation en pédiatrie. L'objectif de cette étude était d'évaluer le profil épidémiologique des pathologies respiratoires chez l'enfant à l'hôpital d'enfants de Rabat, Maroc.

**Méthodes:**

Il s'agit d'une étude observationnelle transversale qui a concerné tous les cas d'enfants âgés de 3 mois à 15 ans hospitalisés pour une pathologie respiratoire au service de pneumoallergologie et infectiologie pédiatriques de l'hôpital d'enfants de Rabat sur une période d'une année, du 1 janvier 2014 au 31 décembre 2014.

**Résultats:**

Sur 3537 patients hospitalisés, 2493 (70,5%) l'ont été pour une pathologie respiratoire. Les hospitalisations pour exacerbation d'asthme (p < 0,001), bronchiolite aigüe (p < 0,001) et dyspnée laryngée (p = 0,004) étaient plus fréquentes chez le garçon alors que les hospitalisations pour pneumopathie aigüe (p = 0,005), pour inhalation de corps étranger (p = 0,007) et pour coqueluche (p = 0,020) étaient plus fréquentes chez la fille. Les hospitalisations pour pneumopathie aigüe (p < 0,001), exacerbation de séquelles graves de virose (p < 0,001) et pour coqueluche (p < 0,001) étaient plus fréquentes chez le nourrisson. Les hospitalisations pour pneumopathie aigüe (p < 0,001) et pour coqueluche (p = 0,015) étaient plus fréquentes en période automnohivernale.

**Conclusion:**

Les motifs d'hospitalisation étaient dominés par les exacerbations d'asthme et la bronchiolite aigüe, lesquelles étaient plus fréquentes chez le garçon. Les infections respiratoires, représentées par les pneumopathies aigües et la coqueluche, étaient plus fréquentes en période automnohivernale et touchaient plus le nourrisson.

## Abstract (en)

**Introduction:**

Respiratory diseases are a common cause of pediatric hospitalization. This study aimed to evaluate the epidemiological profile of respiratory diseases among children at the Rabat Children's Hospital, Morocco.

**Methods:**

We conducted an observational-cross sectional study of all children aged 3 months to 15 years hospitalized for respiratory disease at the Department of Pneumoallergology and Pediatric Infectious Diseases at the Rabat Children's Hospital, Morocco over a one-year period, from 1 January 2014 to 31 December 2014.

**Results:**

Out of 3537 hospitalized patients, 2493 (70.5%) had respiratory disease. Hospitalizations due to asthmatic exacerbation (p < 0.001), acute bronchiolitis (p < 0.001) and laryngeal dyspnoea (p = 0.004) were more frequent among boys, while hospitalizations due to acute pneumonia (p = 0.005), inhalation of a foreign body (p = 0.007) and pertussis (p = 0.020) were frequent among girls. Hospitalizations due to acute pneumonia (p < 0.001), exacerbation of serious viral disease sequelae (p < 0.001) and pertussis (p < 0.001) were more frequent among infants. Hospitalizations due to acute pneumonia (p < 0.001) and pertussis (p = 0.015) were more frequent during the autumn-winter period.

**Conclusion:**

The causes of hospitalization were dominated by asthmatic exacerbations and acute bronchiolitis, which were more frequent among boys. Respiratory infections, such as acute pneumonitis and pertussis, were more frequent during the autumn-winter period and mainly affected the infants.

## Introduction

Les pathologies respiratoires de l'enfant sont un motif fréquent d'hospitalisation en pédiatrie. Dans son rapport de 2015 évaluant les performances du programme national de lutte contre les infections respiratoires aigües chez les enfants âgés de 0 à 59 mois, le ministère de la santé a estimé que 32.251 enfants ont présenté une pathologie respiratoire grave durant l'année 2014 dans les différentes régions du Maroc [1]. Ces pathologies et les hospitalisations qu'elles nécessitent constituent toujours un des principaux problèmes de santé publique au Maroc du fait des couts très élevés qui sont générés. Des données plus complètes et des statistiques plus détaillées sur les hospitalisations ayant pour motif une pathologie respiratoire chez l'enfant sont nécessaires pour comprendre le fardeau global de ces maladies. L'objectif de cette étude a été d'évaluer le profil épidémiologique des pathologies respiratoires chez les enfants hospitalisés à l'hôpital d'enfants de Rabat, Maroc.

## Méthodes

Il s'agit d'une étude observationnelle transversale qui a concerné les cas d'enfants ayant consulté aux urgences médicales pédiatriques puis adressés pour hospitalisation pour une pathologie respiratoire au service de pneumoallergologie et infectiologie pédiatriques de l'hôpital d'enfants de Rabat sur une période d'une année, du 1 janvier 2014 au 31 décembre 2014. Ont été inclus dans l'étude tous les enfants âgés de 3 mois à 15 ans. Les données suivantes ont été recueillies rétrospectivement pour chaque patient depuis le registre des hospitalisations du service de pneumoallergologie et infectiologie pédiatriques: numéro d'entrée du patient, date d'admission, âge, sexe, provenance géographique, diagnostic à l'admission, diagnostic de sortie. Cette étude a été approuvée par le comité d'éthique de la Faculté de Médecine et de Pharmacie de Rabat. La collecte des données était anonyme en utilisant le numéro de dossier des patients. Les données recueillies ont été traitées à l'aide du logiciel IBM^®^ SPSS^®^ Statistics version 23 pour Microsoft^®^ Windows^®^. Le test de khi-deux a été utilisé pour comparer les variables qualitatives. Une valeur p inférieure à 0.05 a été considérée comme statistiquement significative.

## Résultats

Sur 3.537 malades adressés pour hospitalisation au service de pneumoallergologie et infectiologie pédiatriques, 2.493 (70.5%) l'ont été pour une pathologie respiratoire. Le sex-ratio était de 1.6. L'âge des patients variait de 3 mois à 15 ans. Les nourrissons ont représenté 48.1% et les enfants 51.5% de la population étudiée. La majorité des patients provenaient de la région Rabat-Salé-Kénitra (93.8%), les autres patients provenaient de régions plus éloignées du Maroc. Les caractéristiques de la population étudiée sont présentées dans le Tableau 1. Les exacerbations d'asthme représentaient la majorité des cas (51.1%). La deuxième pathologie la plus fréquente était la bronchiolite aigüe (24.4%). Les autres pathologies respiratoires rencontrées sont énumérées dans le Tableau 2. Sur 1.274 hospitalisations pour exacerbation d'asthme, 26 malades (2%) n'étaient pas asthmatiques. Sur 608 nourrissons hospitalisés pour bronchiolite aigüe, 17 (2.4%) ont eu un diagnostic différent à la sortie. Les corrélations entre le diagnostic à l'admission et le diagnostique de sortie des principales pathologies respiratoires rencontrées sont répertoriées dans le Tableau 3. Il existait une différence statistiquement significative entre la fréquence de certaines pathologies respiratoires selon le sexe: les hospitalisations pour exacerbation d'asthme (p < 0.001), bronchiolite aigüe (p < 0.001) et pour dyspnée laryngée (p = 0.004) étaient plus fréquentes chez le garçon alors que les hospitalisations pour pneumopathie aigüe (p = 0.005), pour inhalation de corps étranger (p = 0.007) et pour coqueluche (p = 0.020) étaient fréquentes chez la fille (Tableau 4). Les hospitalisations pour pneumopathie aigüe (p < 0.001), exacerbation de séquelles graves de virose (p < 0.001) et pour coqueluche (p < 0.001) étaient plus fréquentes de façon statistiquement significative chez le nourrisson (Tableau 5). En ce qui concernes les différences selon la saison, les hospitalisations pour pneumopathie aigüe (p < 0.001) et pour coqueluche (p = 0.015) étaient plus fréquentes en période automnohivernale de façon statistiquement significative (Tableau 6).

**Tableau 1:** Caractéristiques de la population^+^

Caractéristiques	Effectif (n = 2493)
Sexe	
Masculin	1546 (62)
Féminin	947 (38)
Age	
Inférieur à 12 mois	559 (22.5)
Entre 12 et 24 mois	650 (26.1)
Entre 3 ans et 5ans Plus de 5	706 (28.3)
ans	578 (23.2)
**Provenance**	
Rabat-Salé-Kénitra	2338 (93.8)
Casablanca-Settat	34 (1.4)
Tanger-Tétouan	57 (2.3)
Fès-Meknès	13 (0.5)
Autres	50 (2)

+ Les valeurs sont exprimées en effectif (pourcentage)

**Tableau 2:** Diagnostics à l’admission^+^

Pathologies	Effectif (n = 2.493)
Exacerbation d’asthme	1274
Bronchiolite aigüe	(51.1)
Pneumopathie aigüe	608 (24.4)
Dyspnée laryngée	271 (10.9)
Inhalation de corps étranger	115 (4.6)
Coqueluche	67 (2.7)
Exacerbation de séquelles graves de	48 (1.9)
virose	32 (1.3)
Autres pathologies	78 (3.12)
Hémoptysie	20
Pleurésie	20
Dilatation des bronches	14
Primo-infection tuberculeuse	12
Hémosidérose	5
Fistule artérioveineuse pulmonaire	3
Abcès pulmonaire	1
Pneumocystose	1
Mucoviscidose	1
Hernie diaphragmatique	1

+ Les valeurs sont exprimées en effectif (pourcentage)

**Tableau 3:** Corrélation entre le diagnostic à l’admission et le diagnostic de sortie^+^

Diagnostic à l’admission	Corrélation avec le diagnostic de sortie	Non corrélation avec le diagnostic de sortie	Diagnostics de sortie non corrélés avec le diagnostic à l’admission
Exacerbation d’asthme	1248 (97.9)	26 (2)	Exacerbation de séquelles graves de virose: 10
			Pneumopathie aigüe: 8
			Dilatation des bronches: 2
			Inhalation de corps étranger: 1
Bronchiolite aigüe	591 (97.2)	17 (2.79)	Dyspnée laryngée: 1
			Pneumopathie aigüe: 3
			Coqueluche: 4
			Exacerbation de séquelles graves de virose: 8
			Double arc aortique: 1
Pneumopathie aigüe	249 (91.8)	22 (8.11)	Primo-infection tuberculeuse: 7
			Séquelles grave de virose: 7
			Malformation adénomatoïde kystique: 2
			Exacerbation d’asthme: 2
			Cardiopathie: 2
			Hernie diaphragmatique: 1
			Bronchiolite aigüe: 1
			Mucoviscidose: 1
Dyspnée laryngée	110 (95.6)	5 (4.34)	Bronchiolite aigüe: 2
			Exacerbation d’asthme: 3
			Inhalation de corps étranger: 1
Inhalation de corps étranger	62 (92.5)	5 (7.46)	Pneumopathie aigüe: 3
			Laryngite virale: 1
			Bronchoscopie négative: 1
Coqueluche	44 (91.6)	4 (8.3)	Bronchiolite aigüe: 2
			Pneumopathie aigüe: 1
			Reflux gastro-œsophagien: 1

+ Les valeurs sont exprimées en effectif (pourcentage)

**Tableau 4:** Motifs d’hospitalisation selon le sexe^+^

Pathologies	Masculin (n = 1546)	Féminin (n = 947)	*p*
Exacerbation d’asthme	804 (52.0)	393 (25.4)	< 0.001
Bronchiolite aigüe	470 (49.6)	215 (22.7)	< 0.001
Dyspnée laryngée	86 (5.6)	29 (3.1)	0.004
Pneumopathie aigüe	147 (9.5)	124 (13.1)	0.005
Inhalation de corps étranger	31 (2.0)	36 (3.8)	0.007
Exacerbation de séquelles graves de virose	20 (1.3)	12 (1.3)	0.955
Coqueluche	22 (1.4)	26 (2.7)	0.020

+ Les valeurs sont exprimées en effectif (pourcentage)

**Tableau 5:** Motifs d’hospitalisation selon la tranche d’âge^+^

Pathologies	Nourrissons (n = 1207)	Enfants (n = 1286)	*p*
Dyspnée laryngée	62 (5.1)	53 (4.1)	0.227
Pneumopathie aigüe	172 (14.3)	99 (7.7)	< 0.001
Inhalation de corps étranger	36 (3.0)	31 (2.4)	0.377
Exacerbation de séquelles graves de virose	25 (2.1)	7 (0.5)	< 0.001
Coqueluche	48 (4.0)	0 (0)	< 0.001

+ Les valeurs sont exprimées en effectif (pourcentage)

**Tableau 6:** Motifs d’hospitalisation selon les saisons^+^

Pathologies	Automne-hiver (n = 1387)	Printemps-été (n = 1106)	*p*
Exacerbation d’asthme	691 (49.8)	583 (52.7)	0.151
Bronchiolite aigüe	332 (23.9)	276 (25.0)	0.556
Dyspnée laryngée	59 (4.3)	56 (5.1)	0.338
Pneumopathie aigüe	178 (12.8)	93 (8.4)	< 0.001
Inhalation de corps étranger	32 (2.3)	35 (3.2)	0.188
Exacerbation de séquelles graves de virose	20 (1.4)	12 (1.1)	0.431
Coqueluche	35 (2.5)	13 (1.9)	0.015

+ Les valeurs sont exprimées en effectif (pourcentage)

## Discussion

Les motifs d'hospitalisation pour pathologie respiratoire chez l'enfant ont été largement dominés dans notre étude par deux pathologies: exacerbation d'asthme qui concerne les enfants (1.274 cas) et la bronchiolite aigüe qui touche le nourrisson (608 cas). Ces pathologies étaient toutes les deux plus fréquentes chez le garçon par rapport à la fille avec respectivement 804 cas versus 393 cas et 470 cas versus 215 cas. D'un autre coté, les infections respiratoires, représentées par les pneumopathies aigües (178 cas versus 93 cas) et la coqueluche (5 cas versus 13 cas), étaient plus fréquentes durant la période automne-hiver par rapport à la période printemps-été. Les pneumopathies aigües touchaient plus le nourrisson que l'enfant (172 cas versus 99 cas) alors la coqueluche n'a concerné que les jeunes nourrissons. Dans notre étude, la grande majorité des patients (93.8%) provenaient du bassin autour de la ville de Rabat, mais certains patients provenaient de régions plus éloignées, probablement en raison de la pénurie des hôpitaux et des infrastructures de santé dans ces régions. Les exacerbations d'asthmes ont représenté 51.1% des motifs d'hospitalisations dans notre étude, alors qu'ils avaient représenté 34% des motifs d'hospitalisation en 2012 dans le même service [2]. Ceci indique que les prévalences de l'asthme de l'enfant et du mauvais contrôle de l'asthme sont en augmentation. Par ailleurs, les exacerbations d'asthme ont été plus fréquentes chez les garçons que chez les filles dans notre étude, ce même résultat a été observé en 2012 dans le même service. Les bronchiolites ont représenté 24.4% des hospitalisations dans notre étude avec une nette prédominance masculine (sex-ratio de 2.18). Un résultat similaire a été retrouvé par Evenou et al. dans une étude réalisée en France entre octobre 2013 et mars 2014 [3]. Nous n'avons par contre pas constaté de différence statistiquement significative entre le nombre de cas de bronchiolite aigüe durant la période automne-hiver et la période printemps-été, alors que Robinson et al ont montré que la saison du virus respiratoire syncytial, responsable de la bronchiolite, débute entre novembre et janvier au Canada et persiste pendant quatre à cinq mois [4]. Certains patients diagnostiqués initialement en tant que bronchiolite aigüe ont vu leur diagnostic redressé en laryngite virale, pneumopathie aigüe, inhalation de corps étranger et exacerbation de séquelles graves de virose. Ces pathologies sont connues pour constituer des diagnostics différentiels de la bronchiolite aigüe [5].

Dans notre étude, les pneumopathies aigües ont représenté 10,9% des hospitalisations, alors qu'une étude réalisée au Mali sur une série de 2.261 enfants hospitalisés a révélé un taux beaucoup plus élevé (68.38%) d'enfants hospitalisés pour pneumopathie aigüe [6]. Le sex-ratio pour les pneumopathies aigües était de 0.72 dans notre étude. Cette prédominance du sexe féminin ne concorde pas avec les résultats d'autres études où il y a une prédominance masculine: le sex-ratio était égal à 1,2 dans une étude réalisée par Nágoan et al en 2012 en Côte d'Ivoire et égal à 1,3 au Togo dans une étude plus ancienne de Bakondé et al [7, 8]. Dans notre étude, les nourrissons étaient plus touchés par les pneumopathies aigües que les enfants, ce qui a été observé dans les études de Nágoan et al et Bakondé et al. Ceci pourrait s'expliquer par les conditions socioéconomiques défavorables dans les pays en voie développement. Dans notre étude, les pneumopathies sont plus fréquentes en période automnohivernale et des résultats similaires ont été observés dans d'autres études [8, 9]. Dans 8 cas d'exacerbation d'asthme et 3 cas de bronchiolite aigüe, le diagnostic retenu à la sortie a été une pneumopathie aigüe. Ceci pourrait être expliqué par l'absence dans certains cas de signes radiologiques évocateurs en cas de pneumopathie communautaire débutante. À l'inverse, 8.11% des diagnostics de pneumopathie aigüe à l'admission se sont avérés être incorrects avec notamment 7 cas de primo-infection tuberculeuse et 7 cas de séquelles grave de virose. Dans notre étude, 4.6% des malades ont été hospitalisés pour dyspnée laryngée avec un sex-ratio de 1.8. Cette prédominance masculine a été observée dans d'autres études: Chobli et al ont constaté un sex-ratio de 1.38 et Sakakura et al ont constaté un sex-ratio de 1,2 [10,11]. Dans notre étude, seuls 4.34% des dyspnées laryngées ont été confondues à l'admission avec une exacerbation d'asthme, une bronchiolite aigüe ou une inhalation de corps étranger. En effet, les signes cliniques de la dyspnée laryngée sont assez évocateurs et le risque de diagnostic erroné à l'admission est par conséquent limité. Dans notre étude, 2.7% des patients ont été hospitalisés pour inhalation de corps étranger et le sex-ratio était de 0.5. Cette prédominance féminine n'a pas été constatée par Diop et al et Vokwely et al qui ont trouvé un sex-ratio respectivement de 2.4 et 2 [12, 13].

Dans notre étude, la coqueluche a représenté 1.9% des hospitalisations avec 4 cas de coqueluche étiquetées initialement comme bronchiolite aigüe. Ceci peut être expliqué par l'existence possible de cas de co-infection par *Bordetella pertussis* et par le virus respiratoire syncytial. L'absence de cas de coqueluche chez l'enfant s'explique par la large couverture vaccinale anticoquelucheuse au Maroc qui fait que seuls les jeunes nourrissons non protégés par les anticorps maternels et non encore complètement vaccinés sont à risque de contracter la maladie [14]. Les exacerbations des séquelles graves de virose ont représenté 1.3% des cas des motifs d'hospitalisation dans notre étude. L'hétérogénéité clinique de cette maladie explique certaines errances diagnostiques. Ainsi dans notre étude, 10 cas d'exacerbation d'asthme, 8 cas de bronchiolite aigüe et 7 cas de pneumopathie aigüe se sont révélé être des exacerbations des séquelles graves de virose après investigation. Certaines limitations de cette étude doivent êtres signalées. Seuls les patients hospitalisés dans le service de pneumoallergologie et infectiologie pédiatriques ont été pris en compte dans cette étude. Les patients hospitalisés dans les autres services de l'hôpital d'enfants de Rabat et les patients ayant consulté au service des urgences médicales pédiatriques et traité en ambulatoire n'ont pas été inclus. Ainsi, les nouveau-nés et les nourrissons de moins de 3 mois sont systématiquement hospitalisés dans d'autres services de l'hôpital d'enfants et les adolescents âgés de plus de 15 ans ne sont pas admis et sont adressés à l'hôpital Avicenne de Rabat. Les patients présentant des formes graves de pathologies respiratoires nécessitant un support respiratoire sont quand à eux hospitalisés dans le service de réanimation pédiatrique. De même, des cas de bronchiolite aigüe du nourrisson peuvent être hospitalisés dans d'autres services de l'hôpital d'enfants notamment en période épidémique. Une autre limitation de cette étude était liée à la collecte des données qui a été réalisée à partir du registre des hospitalisations du service de pneumoallergologie et infectiologie pédiatriques et qui s'est limitée aux paramètres qui y étaient inscrits. Une étude similaire qui se baserait également sur les dossiers des patients et pas uniquement sur le registre du service permettrait d'étudier plus de paramètres.

## Conclusion

Les motifs d'hospitalisation pour pathologies respiratoires chez l'enfant étaient dominés dans notre étude par les exacerbations d'asthme et la bronchiolite aigüe, lesquelles étaient plus fréquentes chez le garçon. Les infections respiratoires, représentées par les pneumopathies aigües et la coqueluche, étaient plus fréquentes en période automnohivernale et touchaient plus le nourrisson. D'autres études plus détaillées sur chacune des pathologies respiratoires de l'enfant sont nécessaires pour obtenir des données épidémiologiques plus précises ce qui pourrait aider à l'élaboration de stratégies de prévention des ces maladies adaptées à notre contexte.

### Etat des connaissances actuelles sur le sujet

- Les pathologies respiratoires représentent un motif fréquent d'hospitalisation en pédiatrie;
- Il n'existe pas à notre connaissance d'études marocaines récentes concernant le profil épidémiologique des hospitalisations pour pathologie respiratoire chez l'enfant.

### Contribution de notre étude à la connaissance

- Cette étude comble une lacune dans la littérature sur le profil actuel des hospitalisations pour pathologie respiratoire chez l'enfant au Maroc;
- Nos résultats pourront aider à l'élaboration de stratégies de prise en charge des pathologies respiratoires chez l'enfant au Maroc.

## Conflits d’intérêts

Les auteurs ne déclarent aucun conflit d'intérêts.
